# Association of food insecurity and sleep difficulty among 189,619 school-going adolescents: a study from the global in-school students survey

**DOI:** 10.3389/fpubh.2023.1212254

**Published:** 2023-07-12

**Authors:** Emmanuel Osei Bonsu, Maxwell Afetor, Lambongang Munkaila, Reforce Okwei, Stephen Uwumbordo Nachibi, Benjamin Noble Adjei, Eric Frimpong, Abdul Wahid Arimiyaw, Collins Adu, Prince Peprah

**Affiliations:** ^1^Department of Epidemiology and Biostatistics, School of Public Health, Kwame Nkrumah University of Science and Technology, Kumasi, Ghana; ^2^Department of Health Information, Ho Polyclinic, Ghana Health Service, Accra, Ghana; ^3^Department of Agribusiness and Applied Economics, North Dakota State University, Fargo, ND, United States; ^4^Department of Geography, Miami University, Oxford, OH, United States; ^5^Department of Geography, Environment and Earth Sciences, University of Hull, Hull, United Kingdom; ^6^Audiology Unit, Kwame Nkrumah University of Science and Technology, Kumasi, Ghana; ^7^Department of Geography and Rural Development, Kwame Nkrumah University of Science and Technology, Kumasi, Ghana; ^8^College of Public Health, Medical and Veterinary Sciences, James Cook University, Townsville, QLD, Australia; ^9^Center for Social Research in Health, UNSW, Sydney, NSW, Australia; ^10^Social Policy Research Centre, UNSW, Sydney, NSW, Australia; ^11^Centre for Primary Health Care and Equity, UNSW, Sydney, NSW, Australia

**Keywords:** food insecurity, sleep disturbance, adolescents, global health, multinomial

## Abstract

**Introduction:**

Adolescents’ sleep disturbances are associated with chronic and dramatic physical, emotional, and mental development and school performance consequences. Although food insecurity could significantly contribute to these effects, few studies have explored the effect of food insecurity on sleep disturbances among adolescents. The study aimed to examine the relationship between adolescents’ food insecurity and sleep disturbance.

**Methods:**

Data on 189,619 adolescents were drawn from the cross-sectional global adolescent health surveys conducted between 2015 and 2018 in 35 countries and territories. Univariate and multivariable multinomial regression models were fitted to examine the hypothesized associations.

**Results:**

Overall pooled prevalence of moderate [45.2% (95%CI = 43–47)] and severe [5.8% (95%CI = 5–6)] food insecurity levels were reported. About [52.6% (95%CI = 51–54)] moderate and [8.6% (95%CI = 8–9)] severe worry-induced sleep disturbances were found. Considering the fully adjusted multinomial logistic model, moderate food insecurity was significantly associated with moderate (AOR = 1.70 CI = 1.59–1.81; *p* < 0.0001) and severe (AOR = 1.63 CI = 1.42–1.87; *p* < 0.0001) sleep disturbances. Also, adolescents reporting severe levels of food insecurity had moderate (AOR = 1.88 CI = 1.68–2.11; *p* < 0.0001) and severe (AOR = 4.07 CI = 4.74–6.11; *p* < 0.0001) sleep disturbances. Females and those aged between 15 and 17 years and 18 or more were at higher risk of moderate and severe sleep disturbances in the context of food insecurity.

**Conclusion:**

Reducing food insecurity could be an effective policy strategy for enhancing adolescent sleep quality.

## Introduction

Recent estimates of access to food present a troubling picture of global food insecurity. For instance, in 2020, between 720 and 811 million people across the globe were estimated to have faced hunger (1). This report presents a substantial problem in attaining the United Nations’ Global Goals for Sustainable Development, which call for the global eradication of extreme hunger and all types of malnutrition by 2030 (1). Food insecurity is defined as the interruption of food consumption or patterns of eating due to a lack of money and other resources [(2), p. 27]. Although food insecurity is not limited to only developing countries, a large proportion comes from the developing world. For example, between 2019 and 2020, developing countries accounted for over 80% of the sharp increase in food insecurity (3). Thus, food insecurity is a major global issue affecting developing and developed countries (4).

Among adolescents and children, food insecurity is associated with poor academic performance due to learning difficulties, nutrient deficiencies, and poor outcomes in terms of physical health and mental health (5–8). Food insecurity is strongly linked with sleep through psychological distress, depression, anxiety, and hunger disturbance, especially among adolescents (9–11). For instance, among adolescents from 68 countries, Wang (11) established that severe food insecurity increases the risk of sleep disturbance in 48 countries. Lee et al. (9) also reported that adolescents in the persistently low food insecurity group and persistently moderate food insecurity group faced more sleep difficulties than those in the food-secure group in Taiwan. Even beyond adolescents, previous studies have found that food insecurity impacts sleep negatively among older adults (2, 10, 12, 13).

Poor sleep quality and sleep disturbances are significant public health concerns (2, 14). Chronic poor-quality sleep is associated with numerous adverse health outcomes such as psychological distress, all-cause mortality, type 2 diabetes, obesity, and chronic cardiovascular diseases (14–17), stress, depression, and anxiety (10).

With the recent increase in food insecurity (3), heightened sleep disturbances and related adverse health outcomes are likely, especially among adolescents (11, 18). Reports from previous studies show that the proportion of adolescents who are food insecure is higher than other subpopulation groups, and one-third of adolescents across the globe experience sleep difficulties (11, 16, 19). Examining the association between food insecurity and sleep is essential for public health and policy intervention for sleep disturbance-related disease burden control. However, published studies on the association between food insecurity and sleep are limited, especially among adolescents (11). The limited literature, however, predominantly focuses on the adult and aged populations (2, 10, 12, 13). Few studies that examined adolescent sleep disturbances have only focused on the mental health implications (4, 9, 20).

To the best of our knowledge, the only study examining food insecurity and sleep disturbances among adolescents using data from multiple country surveys was conducted by Wang (11). However, Wang’s (11) study has three significant limitations requiring further analysis. For instance, Wang’s study used relatively old Global School-Based Student Health Surveys’ (GSHS) data. Some of the data included in the analysis were collected in 2007, which is more than a decade ago and might not reflect the recent or current reality of food insecurity and sleep issues. The present study uses data from surveys conducted between 2015 and 2018, which remain the most current GSHS data in the countries included. More importantly, previous findings have indicated that the age and sex of an individual may differ in health status and also predict psychological state, including sleep patterns (21), which was not examined in Wang’s study. Age and sex are important determinants of health because of the biological, social, economic, psychological, and behavioral changes attributed to them (22). For instance, increasing age declines physical health, whereas stress levels, psychological health, and social health have been found to also increase with age (23). Meanwhile, the differential vulnerability hypothesis (24) suggests that exposure to social health determinants such as food insecurity remains varied. For instance, age and gender differences may relate differently, mainly due to differences in social and economic deprivations (2). Furthermore, as the adolescent population includes more females than males, sex and age are critical determinants for food insecurity and sleep. Again, potential biological factors such as hormonal changes and genetic factors may also have a negative impact on the relationship between food insecurity and sleep quality (14). Lastly, Wang’s study measured food insecurity and sleep disturbances using dichotomous variables and analyzed them through a binary regression approach. However, using such an approach appears simplistic. Food insecurity has been widely categorized into moderate and severe levels (4, 25). Moderate food insecurity indicates that the quantity/quality of food intake has been compromised, and severe food insecurity points to decreased food consumed and disrupted eating patterns (7). These categories, therefore, represent a better operationalization of food insecurity (7).

Thus, using a multinomial logit analytical approach, this study intended to examine whether food insecurity is associated with sleep disturbances among a representative sample of 189,619 adolescents from 35 countries and territories using the most recent GSBHS data. We also examine the modifying roles of age and sex in the association between food insecurity with sleep disturbance. We hypothesized that (1) adolescents who are moderately and severely food-insecure would have increased risks of sleep disturbance and (2) females and increasing age also increase the likelihood of sleep disturbance.

## Methods

### Data source

This cross-sectional, multi-country study used publicly available data from the GSHS among 35 countries. The GSHS is a representative and extensive health survey of risk factors and behaviors of students. US Centers for Disease Control and Prevention (CDC) and the World Health Organization (WHO), and other United Nations (UN) allies and country-specific institutions developed this survey (1, 26). Content from this survey was drawn from the CDC Youth Risk Behaviors Survey (YRBS) to establish its reliability and validity (27). The information included in the survey is the objective, methodology used in the survey, and the procedure for sampling the GSHS is available at http://www.cdc.gov/gshs/.

In summary, though the participants for the GSHS are primarily school-going adolescents aged 13–17 years, those below 13 and above 17 years were also included in the survey. The sampling procedure included a two-stage standardized probability sampling approach for selecting participants within each country in this survey. In the first stage, the schools were selected through probability proportional to size sampling design. In the second stage, students between the ages of 13 and 17 were randomly selected from their various classrooms in each selected school. Irrespective of the student age in the selected classrooms, all students were eligible to participate in the survey. In this study, we employed the most current data available in GSHS participating nations and territories in this investigation. Countries and territories with data released before 2015 were removed from the analysis. These criteria ensured that the analysis reflects and represents recent or current trends. Also, we excluded data that did not have the variables of interest for the present study. By applying the inclusion and exclusion criteria, data from 35 countries and territories were considered recent and eligible to be included in the analysis. Thus, our analysis included data from 35 countries and territories released between 2015 and 2018. To account for non-response and probability selection, the data was weighted to enable the generalization of results to the targeted population (28, 29).

### Data collection procedure

Trained enumerators collected data from local social work agencies and other research institutions during a regular class period. The GSHS questionnaire was designed to be close-ended and was written in English before being translated into each country’s native language. Multiple-choice questions were included in the questionnaire. The questionnaires were administered after informed consent was obtained from children, parents, and school authorities. Before the questionnaires were given, the trained enumerators thoroughly described the purpose of the study and the instructions to the students.

Furthermore, through anonymous and voluntary participation, the survey ensured that students’ privacy was respected. The study followed the Helsinki Declaration, and all GSHS surveys were evaluated and approved by institutional review boards or ethics committees in each nation. Details of the systematic techniques used to gather student data can also be obtained at http://www.cdc.gov/gshs/.

### Assessing food insecurity (hunger)

We assessed food insecurity (hunger) by asking, “During the past 30 days, how often did you go hungry because there was not enough food in your home?” The item had 5-point response options: never, rarely, sometimes, most of the time, and always. It has been argued that single-item measures can be easier to capture and more cost-effective in large-scale surveys like the GSHS than multiple-item measures (30). A single item should be sufficient for the variable, and the attribute of the variable measured is “concrete” to the participants (31). We believe the single item assessing food insecurity in the GSHS was an example of such a concrete measure. Given the exploratory nature of the present study, we believed that the single-item question was a sufficient measure of food insecurity, consistent with previous studies (4, 17, 32, 25). For analytic purposes and in line with other previous studies, we categorized never as no food insecurity, rarely and sometimes as moderate food insecurity and most of the time and always as severe food insecurity. These categories were named as such because evidence suggests that moderate food insecurity is often considered to indicate that the quality/quantity of food intake has been compromised. In contrast, severe food insecurity refers to reduced food intake and disturbed eating patterns (7).

### Assessing sleep disturbances

Sleep disturbance was assessed based on the question, “During the past 12 months, how often have you been so worried about something that you could not sleep at night?” Responses were never, sometimes, most of the time, and always. This item has been used in previous global studies involving adolescents using the GSHS (11, 33). In this study, never was categorized as no sleep disturbance, sometimes as moderate sleep disturbance, and most of the time and always as severe sleep disturbance for analytic purposes.

### Covariates

We included empirically and theoretically defined variables as confounders. These covariates were selected because they both have direct and indirect relationship with food insecurity and sleep quality in previous studies (4, 17, 32, 34, 25). Sociodemographic variables included age (11–14 years; 15–17 years; 18+ years) and sex (male; female). Health-related and behavior variables included bullying victimization (no; yes), loneliness (no; yes), suicidal ideation (no; yes), cigarette smoking (no; yes), alcohol use (no; yes), marijuana use (no; yes), physical activity (no; yes), close friends (no; 1–2; 3+), amphetamine use (no; yes), and parents understand adolescent problems (no; yes).

### Statistical analysis

Stata 14.0 was used to perform the statistical analysis. The ‘svyset’ command in Stata was used to adjust for the complex sampling design employed by the GSHS survey using the weight, primary sampling unit (psu) and stratum variables. A *p*-value ≤0.05 was used to assess the statistical significance. Frequencies and percentages were calculated to describe the characteristics of respondents. Country-specific prevalence of food insecurity and sleep disturbance were summarized using proportions and 95% confidence intervals by sex. A series of stepwise multinomial logit regression analysis was performed to assess the independent effect of food insecurity on sleep disturbance. Three separate models were fitted to estimate the association. Model 1 calculated the crude regression estimates by predicting the effect or influence of food insecurity on sleep quality. In Model 2, we added sociodemographic variables that could predict food insecurity or sleep disturbance. In Model 3 (full model), we included covariates related to lifestyle, support, and health-related variables. Further, moderation analyses of age and sex were performed to examine their modifying roles in the association between food insecurity and sleep disturbance.

## Result

### Background characteristics of respondents

A total of 189,619 adolescents from 35 countries and territories were involved in the study. Females (50.4%) were more than the males. The majority, 53.8% of adolescents, were between 15 and 17. The majority (66.4%) of adolescents were not bullied; 65.4% were lonely; 89.6% had no suicidal ideas; 74.3% had more than three close friends; 88.8% had not used cigarettes; 83.8% had not used alcohol; 96.2% had not used marijuana; 96.8% had not used amphetamine; 68.6% were physically active, and 77% had parents who understood their problems (Table 1).

**Table 1:** Distribution of relevant variables among adolescents in 35 countries.

Variable	*N* = 189,619 (%)	Weighted % (95% CI)
Sex		
Male	90,054	49.6 (50.3–52.5)
Female	99,565	50.4 (48.2–50.4)
Age		
11–14 years	84, 418	41.6 (39.5–44.6)
15–17 years	95,512	53.8 (50.8–57.5)
18+ years	9,260	4.6 (4.2–5.1)
Bullied		
No	119,285	66.3 (65.6–68.7)
Yes	61,114	33.7 (32.4–35.8)
Loneliness		
No	66,439	34.6 (34.2–36.6)
Yes	120,799	65.4 (64.5–66.3)
Suicidal Ideation		
No	153,483	89.6 (89.5–90.6)
Yes	26,304	10.4 (10.3–11.8)
Close friend		
No	3,945	5.1 (5.5–6.5)
1–2	47,479	20.6 (19.2–22.8)
3+	126,582	74.3(72.7–76.1)
Cigarette Use		
No	147,923	88.8 (88.7–90.2)
Yes	24,789	11.2 (10.5–12.3)
Alcohol Use		
No	100,442	83.8 (83.2–85.8)
Yes	49,681	16.2 (15.0–17.5)
Marijuana Use		
No	5,401	96.2 (96.2–97.7)
Yes	8,188	3.8 (3.5–4.6)
Amphetamine Use		
No	156,364	96.8 (96.1–97.3)
Yes	4,713	3.2 (3.0–4.1)
Physical Activity		
No	42,469	31.4 (30.5–33.9)
Yes	128,388	68.6 (67.7–70.8)
Parents understand adolescent problems		
No	39,821	23.0 (22.8–24.6)
Yes	125,254	77.0 (76.2–78.5)

### Prevalence of food insecurity and sleep disturbance among adolescents

Tables 2, 3 present the overall and country-specific prevalence of food insecurity and sleep disturbance, respectively. The overall pooled prevalence of food insecurity (moderate and severe) was 51%. Food insecurity was more common among males (52%) than females. Overall pooled prevalence of sleep disturbance (moderate and severe) was 61.2%. Unlike food insecurity, sleep disturbance was more common among female adolescents (64.9%) than male adolescents (see Table 2). Regarding gender, the highest prevalence of food insecurity among male adolescents was reported in Samoa (75.7%), and the lowest prevalence was found in Curacao (25%). Among females, the highest prevalence of food insecurity was reported in Samoa (72.3%), and the lowest was found in Curacao (25%). Regarding sleep disturbance, the highest among males was reported in the Philippines (73.3%), and the lowest was observed in Suriname (44.9%). Among females, the highest was reported in the Philippines (79.7%), and the lowest was seen in Myanmar (47.2%; see Table 3).

**Table 2:** Overall pooled prevalence of food insecurity and sleep disturbance among adolescents in 35 countries.

Food Insecurity	Frequency *N* (%)	Overall Prevalence	Males (95% CI)	Females (95% CI)
No food insecurity	105,286 (55.98)	49.0 (48.2–51.5)	47.7 (46.5–49.8)	51.5 (50.1–53.2)
Moderate food insecurity	72,028 (38.30)	45.2 (43.0–47.3)	46.3 (45.4–48.6)	43.8 (42.3–46.4)
Severe food insecurity	10,752 (5.72)	5.8 (5.2–6.7)	6.0 (5.1–7.6)	4.7 (4.7–5.6)
Sleep disturbance				
No sleep disturbance	66,089 (35.18)	38.8 (38.0–40.3)	42.5 (41.3–44.0)	35 (33.5–36.8)
Moderate sleep disturbance	99,117 (52.76)	52.6 (51.9–54.5)	49.8 (48.8–51.0)	55.4 (54.5–57.3)
Severe sleep disturbance	22,666 (5.37)	8.6 (8.8–9.5)	7.8 (7.8–8.9)	9.5 (8.3–10.4)

**Table 3:** Country-specific prevalence of food insecurity and sleep disturbance among adolescents in 35 countries by sex.

Country		Food Insecurity						Sleep disturbance					
	Frequency	No food insecurity		Moderate food insecurity		Severe food insecurity		No sleep disturbance		Moderate sleep disturbance		Severe Sleep disturbance	
	*N* (%)	Males % (95% CI)	Female % (95% CI)	Males % (95%CI)	Female % (95% CI)	Males % (95%CI)	Female % (95% CI)	Males % (95% CI)	Female % (95% CI)	Males % (95% CI)	Female % (95% CI)	Males % (95% CI)	Female % (95% CI)
Anguilla	812 (0.44%)	58.1(53–63)	55.9(50–61)	35.3(30–40)	39.9(34–45)	6.6(5–9)	4.2(3–7)	49.3(43–55)	28.9(25–34)	43.6 (38–49)	58.6(54–64)	7.1(5–10)	12.4 (9–16)
Argentina	56,873 (31.11%)	68.4 (66–69)	68.5(67–70)	29.3(28–31)	29.7(28–31)	2.3(1–2)	1.7 (1–2)	38.1 (37–23)	21.6 (20–23)	53.8(52–55)	60.4(59–62)	8.1 (7–8)	18.0 (17–19)
Bahrain	7,137 (3.90%)	43.5 (40–47)	43.4(39–48)	45.6(42–48)	45.2 (41–49)	10.9(9–13)	11.4 (10–13)	41.6 (38–44)	21.8(19–25)	47.26 (44–51)	56.5(54–58)	11.2(10–13)	21.7(18–25)
Benin	2,535 (1.39%)	39.0(33–46)	50.9(44–58)	41.4(37–46)	34.0(30–39)	19.6(15–25)	15.0(11–20)	26.7(24–30)	28.9(24–34)	52.0(48–55)	51.3(45–57)	21.2(18–25)	19.8(16–24)
Cooks Island	689 (0.38%)	31.6(26–38)	28.7(23–35)	59.5(54–65)	60.3(52–68)	8.8(6–13)	11.0(7–17)	44.4(38–50)	24.4(20–29)	46.5(41–52)	55.9(51–61)	9.2(6–13)	19.6(16–24)
Curacao	2,755 (1.51%)	74.9(72–77)	75.0(73–77)	21.1(19–24)	21.8(20–24)	3.9(3–6)	3.2(2–4)	53.0(49–56)	30.2(27–34)	39.5(36–43)	55.1(51–59)	7.5(6–9)	14.7(13–17)
Dominican Republic	1,464 (0.80%)	62.0(54–69)	66.6(59–73)	35.4(28–44)	30.7(25–38)	2.5(1–5)	2.6(2–5)	42.8(37–48)	29(23–35)	50.2(45–55)	58.0(53–62)	7.0(4–10)	13.4(10–18)
Fiji	3,673 (2.01%)	41.0(36–46)	39.2(34–45)	48.5(45–52)	48.9(45–52)	10.5(8–13)	11.9(10–14)	45.6(41–51)	38.8(34–44)	42.0(39–45)	47.1(43–51)	12.5(9–16)	14.1(13–16)
French Polynesia	3,210 (1.76%)	37.5(34–41)	32.9(30–36)	52.1(49–55)	55.4(53–58)	10.4(8–13)	11.6(10–14)	45.5(43–48)	32.2(29–35)	46.0(42–50)	52.5(50–55)	8.5(7–10)	15.3(14–17)
Guatemala	4,319 (2.36%)	60.0(55–64)	65.4(58–72)	37.7(33–43)	31.0(25–37)	2.6(1–6)	3.6(2–6)	47.8(43–53)	34.6(30–40)	47.3(42–53)	56.4(52–61)	5.0(3–7)	8.9(7–12)
Indonesia	11,124 (6.09%)	42.6(40–45)	46.7(43–50)	52.7(50–55)	49.6(46–53)	4.6(4–6)	3.7(3–4)	51.0(48–54)	42.8(39–46)	44.0(41–47)	53.0(50–56)	5.0(4–6)	4.2(4–5)
Jamaica	1,659 (0.91%)	53.3(46–60)	47.8(44–52)	38.8(33–45)	46.2(43–50)	7.9(6–9)	6.0(4–8)	49.9(44–56)	35.1(30–41)	41.2(36–47)	48.2(44–52)	8.8(7–11)	16.7(14–20)
Kuwait	3,605 (1.92%)	52.5(49–55)	49.8(45–54)	41.2(39–43)	40.9(37–45)	6.3(5–9)	9.3 (8–11)	34.0(31–37)	20.5(18–23)	50.4(47–54)	53.0(49–57)	15.6(12–19)	26.5(23–31)
Lao Republic	3,633 (1.99%)	48.1(41–55)	53.3(47–59)	51(44–57)	45.5(40–52)	1.1(0.4–3)	1.2(0.5–3)	37.9(35–41)	27.9(25–31)	57.9(55–61)	66.1(63–69)	4.1(3.0–5.6)	6.0(5–7)
Lebanon	5,692 (3.11%)	69.1(66–72)	69.2(67–72)	27.4(24–31)	27.6(25–30)	3.5(3–5)	3.2(3–4)	41.6(39–44)	26.2(23–29)	48.7(46–52)	56.6(54–59)	9.6(8–12)	17.2(15–20)
Liberia	2,661 (1.46%)	31.0(27–35)	33.0(27–40)	50.4(46–55)	52.3(47–58)	18.5(15–22)	14.7(12–19)	28.6(25–33)	22.1(19–25)	53.5(50–57)	56.3(52–60)	17.9(15–21)	21.5(19–24)
Mauritius	3,009 (1.65%)	56.4(52–61)	57.0(51–63)	38.4(35–42)	33.5(29–38)	5.2(4–6)	10.0(6–15)	47.9(44–52)	31.2(26–36)	44.7(40–49)	58.0(54–62)	7.5(6–9)	10.8(9–13)
Morocco	6,633 (3.63%)	62.9(60–66)	69.5(67–72)	27.5(24–31)	22.1(20–24)	9.6(8–12)	8.4(7–10)	45.4(42–48)	32.8(29–37)	40.7(37–45)	47.0(44–50)	13.8(12–16)	20.2(18–22)
Mozambique	1,889 (1.03%)	47.9(39–57)	55.5(46–64)	40.2(33–48)	34.1(26–43)	11.9(9–15)	10.5(7–14)	41.0(37–45)	34.5(27–43)	50.3(46–5)	53.3(45–61)	8.7(6–13)	12.2(8–17)
Myanmar	2,828 (1.55%)	66.1(63–69)	71.2(68–74)	31.0(29–34)	26.9(24–30)	2.9(2–5)	2.0(1–3)	54.9(52–58)	52.8(49–56)	41.8(39–45)	43.4(40–46)	3.4(3–4)	3.8(3–5)
Nepal	6,481 (3.55%)	67.3(62–72)	66.5(61–71)	27.9(24–32)	29.1(25–34)	4.8(3–8)	4.3(2–9)	50.9(48–54)	50.1(47–54)	44.4(42–47)	45.8(43–49)	4.7(4–6)	4.1(3–6)
Oman	3,456 (1.89%)	60.8(57–65)	63.1(59–67)	33.3(30–37)	33.2(30–37)	5.9(5–8)	3.7(3–5)	36.9(34–53)	20.0(17–23)	49.9(47–53)	56.9(53–61)	13.2(12–15)	23.2(21–26)
Paraguay	3,101 (1.70%)	69.3(67–71)	72.0(68–76)	27.9(26–30)	26.1(22–30)	2.8(2–4)	1.9(1–3)	40.6(36–45)	28.4(24–33)	52.6(49–56)	59.7(56–63)	6.8(5–8)	11.9(10–14)
Philippines	8,756 (4.79%)	28.3(26–31)	31.5(29–34)	63.5(62–65)	61.6(59–64)	8.2(7–10)	6.8(6–8)	26.7(24–29)	20.4(19–22)	63.9(62–66)	67.1(65–69)	9.4(8–11)	12.6(11–14)
Samoa	1,924 (1.05%)	24.3(20–29)	27.7(23–32)	63.1(58–68)	60.0(56–64)	12.6(9–17)	12.3(9–16)	46.0(40–52)	42.3(39–46)	44.5(39–50)	48.7(46–51)	9.5(7–12)	8.9(7–11)
Seychelles	2,538 (1.39%)	57.1(53–61)	55.2(51–59)	29.0(26–32)	33.7(31–37)	13.8(11–17)	11.1(9–14)	47.7(44–51)	30.9(28–34)	43.3(40–47)	55.8(53–58)	9.0(7–11)	13.3(11–15)
Sri Lanka	3,261 (1.78%)	68.3(63–73)	74.8(71–79)	28.6(24–33)	22.2(19–26)	3.2(2–4)	2.9(2–4)	52.2(48–56)	58.0(53–62)	43.4(40–47)	36.9(33–41)	4.4(3–7)	5.1(4–6)
Suriname	2,119 (1.16%)	59.2(53–65)	52.1(47–58)	30.4(25–36)	34.8(31–39)	10.5(8–13)	13.1(11–15)	55.1(51–59)	38.5(34–43)	36.1(33–39)	45.6(42–49)	8.8(7–11)	15.9(13–19)
Thailand	5,877 (3.21%)	43.6(40–47)	48.0(44–52)	51.9(49–55)	48.7(45–52)	4.6(3–6)	3.3(3–4)	37.6(34–42)	30.1(26–34)	53.3(49–57)	61.2(58–64)	9.1(7–11)	8.7(7–11)
Timor-Leste	3,630 (1.99%)	48.1(45–51)	49.3(46–52)	39.8(37–43)	39.9(37–43)	12.1(10–14)	10.8(9–13)	43.8(39–49)	47.8(4353)	43.0(39–48)	41.0(37–45)	13.2(11–16)	11.1(9–14)
Trinidad and Tobago	3,858 (2.11%)	46.9(42–52)	51.4(47–55)	43.6(3948)	41.9(38–46)	9.6(8–12)	6.7(6–8)	49.2(46–52)	35.4(32–39)	40.1(38–43)	47.9(45–51)	10.8(9–13)	16.6(14–19)
Tonga	3,328 (1.82%)	28.6(26–32)	39.4(36–43)	59.2(56–62)	50.7(48–54)	12.2(10–14)	9.9(8–12)	28.6(26–32)	39.4(36–43)	59.2(56–62)	50.7(48–54)	12.2(10–14)	9.9(8–12)
UAE	5,826 (3.19%)	54.3(50–59)	51.8(48–56)	37.8(35–41)	38.2(35–41)	7.9(6–10)	9.9(8–12)	40.2(36–44)	24.9(22–28)	47.9(44–52)	54.5(52–57)	11.9(10–14)	20.5(18–23)
Vanuatu	2,136 (1.17%)	34.3(30–39)	42.4(38–47)	56.2(52–61)	50.9(47–55)	9.5(7–12)	6.6(5–8)	45.1(41–49)	43.9(39–49)	48.7(45–53)	48.5(45–52)	6.1(5–8)	7.5(6–10)
Wallis and Futuna	1,110 (0.61%)	36.5(32–41)	35.8(31–41)	50.0(46–54)	48.3(44–52)	13.4(10–17)	15.9(13–20)	43.4(39–48)	34.9(30–40)	43.1(38–48)	47.3(42–53)	13.4(11–17)	17.8(15–21)

### Association between food insecurity and sleep

Table 4 shows a series of multinomial regressions predicting sleep disturbance. Model 1 estimated the crude effect of the association, whilst Models 2 and 3 controlled for sociodemographic, health and lifestyle-related variables. In Model 1, adolescents who were moderately food insecure relative to those who were food secure were 2.09 times more likely to have a moderate sleep (AOR = 2.09 CI = 1.98–2.20) and severe (AOR = 1.90 CI = 1.71–2.12) sleep disturbances. Similarly, adolescents who were severely food insecure were 2.28 and 5.38 more likely to experience moderate (AOR = 2.28 CI = 2.10–2.48) and severe (AOR = 5.38 CI = 4.74–6.11) sleep disturbances, respectively, relative to their food secure counterparts.

**Table 4:** Multinomial regression models estimating the effect of food insecurity on sleep disturbance among adolescents in 35 countries.

	Model 1		Model 2		Model 3	
Variable	OR (95% C.I)	*p*-value	OR (95% C.I)	*p*-value	OR (95% C.I)	*p*-value
No sleep disturbance (Base outcome)						
Moderate sleep disturbance						
Food security	**1**		**1**		**1**	
Moderate food insecurity	2.09 (1.98–2.20)	**<0.001**	2.11(2.00–2.23)	**<0.001**	1.70 (1.59–1.81)	**<0.001**
Severe food insecurity	1.90 (1.71–2.12)	**<0.001**	1.91 (1.72–2.13)	**<0.001**	1.63 (1.42–1.87)	**<0.001**
Severe sleep disturbance						
Food security	**1**		**1**		**1**	
Moderate food insecurity	2.28 (2.10–2.48)	**<0.001**	2.31 (2.12–2.51)	**<0.001**	1.88 (1.68–2.11)	**<0.001**
Severe food insecurity	5.38 (4.74–6.11)	**<0.001**	5.23 (4.62–5.92)	**<0.001**	4.07 (3.40–4.87)	**<0.001**

*p*-value = ****0.0001, C.I = confidence interval, RR = relative risk. Model 1: Unadjusted model. Model 2: Adjusted for sociodemographic characteristics (age, sex). Model 3: Adjusted for sociodemographic characteristics and other relevant variable (age, sex, loneliness, suicidal ideation, cigarette smoking, alcohol use, close friends, parental understanding, physical activity). Bold values are significant values.

In Model 2, there was a slight change for each outcome, indicating a marginal role of the sociodemographic factors in explaining the outcome variables. Model 3 was adjusted for lifestyle, health, and health-related variables (Full model). The results showed that adolescents who were moderately food insecure relative to those who were food secure were 1.70 and 1.63 more likely to experience moderate (AOR = 1.70 CI = 1.59–1.81) and severe (AOR = 1.63 CI = 1.42–1.87) sleep disturbances, respectively. Likewise, adolescents who were severely food insecure were 1.88 and 4.07 more likely to experience moderate (AOR = 1.88 CI = 1.68–2.11) and severe (AOR = 4.07 CI = 3.40–4.87) sleep disturbances, respectively, relative to their food secure counterparts (see Table 4).

### Age- and sex-wise associations of food insecurity status with sleep disturbance

As displayed in Table 5, moderation analysis was performed to determine the modifying effect of age and sex on the association of food insecurity and sleep disturbance. The age-wise analysis used the interaction effect of age to determine the association between food insecurity and sleep disturbance. There was a significant association between moderate food insecurity and moderate sleep disturbance among adolescents aged 15–17 (AOR = 3.39 CI = 3.39–3.93) and a similar association between moderate food insecurity and severe sleep disturbance among adolescents aged 18 and older (AOR = 3.24 CI = 2.40–4.38). Moreover, there was a significant association between severe food insecurity and severe sleep disturbance among adolescents aged 15–17 (AOR = 1.15 CI = 0.97–1.33), severe food insecurity and severe sleep disturbance among adolescents aged 15–17 (AOR = 1.75 CI = 1.45–2.06), moderate food insecurity and severe sleep disturbance among adolescents aged 18 or older (AOR = −1.60 CI = 0.81–1.24). With sex-wise analysis, the interaction effect of sex was used to assess the association between severe food insecurity and severe sleep disturbance. There was an association between moderate food insecurity and severe sleep disturbance among females (AOR = 2.77 CI = 2.30–3.33), severe food insecurity and severe sleep disturbance among females (AOR = 8.58 CI = 6.95–10.57; see Table 5).

**Table 5:** Age- and sex-wise associations of food insecurity status with sleep disturbance among adolescents in 35 countries.

Variable	Model 1		Model 2	
	OR (95% C.I)	*p*-value	OR (95% C.I)	*p*-value
No Sleep disturbance (Base outcome)				
Moderate sleep disturbance				
Age				
11–14	**1**			
15–17	1.75 (1.63–1.88)	**<0.001**		
18+	2.39 (2.01–2.82)	**<0.001**		
Food Insecurity				
Food Security	**1**		**1**	
Moderate food insecurity	2.08 (**1**.93–2.24)	**<0.001**	2.12 (2.01–2.24)	**<0.0001**
Severe food insecurity	2.07 (1.78–2.41)	**<0.001**	1.97 (1.77–2.20)	**<0.0001**
Age * food security				
11–14 * food security	**1**			
15–17 * moderate food insecurity	3.64 (**3**.39–3.93)	**<0.001**		
15–17 and severe food insecurity	2.89 (**2**.50–3.34)	**<0.001**		
18+ * moderate food insecurity	4.23 (**3**.55–5.04)	**<0.001**		
18+ and severe food insecurity	3.24 (**2**.40–4.38)	**<0.001**		
Sex				
Males			**1**	
Females			1.41 (1.33–1.49)	**<0.001**
Sex * food insecurity				
Females * no food insecurity			**1**	
Females * moderate food insecurity			2.99 (2.75–3.25)	**<0.001**
Females * severe food insecurity			2.77 (2.30–3.33)	**<0.001**
Severe sleep disturbance				
Age				
11–14	**1**			
15–17	0.77 (0.66–0.89)	**<0.001**		
18+	1.48 (**1**.28–1.68)	**<0.001**		
Food Insecurity				
Food security	**1**		**1**	
Moderate food insecurity	0.81 (0.73–0.89)	**<0.001**	2.33 (2.15–2.53)	**<0.001**
Severe food insecurity	1.61 (1.48–1.73)	**<0.001**	5.63 (4.96–6.41)	**<0.001**
Age * food security				
12–14 * food security	**1**			
15-17*moderate food insecurity	1.15 (0.97–1.33)	**0.0001**		
15-17*severe food insecurity	1.75 (1.45–2.06)	**0.0001**		
18+ * moderate food insecurity	1.60 (0.81–1.24)	**0.0001**		
Sex				
Males			**1**	
Females			1.58 (**1**.46–1.71)	**<0.001**
Sex* food security				
Females* food security			**1**	
Females*moderate food insecurity			3.74 (3.31–4.23)	**0.0001**
Females*severe food insecurity			8.58 (6.95–10.57)	**0.0001**

*p*-value was considered significant when less than 0.05, CI = confidence interval, OR = odds ratio. Model 1: Moderation effect of age on food insecurity and sleep difficulty. All Models adjusted for sociodemographic characteristics and other relevant variables such as age, sex, bullying, loneliness, suicidal ideation, cigarette smoking, alcohol use, marijuana use, close friends, amphetamine use, parental understanding, and physical activity.

## Discussion

### Main findings

In this multi-country analysis, 45 and 5.3% were reported to be moderately and severely food insecure, respectively. Similarly, 52.5 and 8.6% of moderate and severe sleep disturbances were found. Adolescents with moderate and severe food insecurity were at higher risk of sleep disturbance than food-secure adolescents. The association between food insecurity and sleep disturbance was evident after adjustments for multiple potential variables. In addition, the effect of food insecurity on sleep disturbance was found in female adolescents. Also, adolescents aged 15–17 years and 18 years or older were found to have an increased effect of food insecurity on sleep than those aged 11–14 years. The study indicates that interventions for quality sleep among adolescents should include ensuring adequate food security.

### Findings interpretation

The study contributes to a growing area of research that broadens understanding of food insecurity and adolescent sleep disorders. Overall, the prevalence of moderate (45.2%) and severe (5.8%) food insecurity were found. Thus, our study’s food insecurity prevalence was within the range of previously documented estimates in multi-country studies (4, 35). The threats of global environmental change in food production systems, sky-rocketing food prices, and natural (and human-induced) disasters could be impacting food insecurity (hunger) prevalence (36). Also, about 52.6% moderate and 8.6% severe sleep disturbances were found, consistent with the rates reported in one multi-country analysis (11). The high prevalence of sleep disturbance among adolescents is troubling because studies have associated poor sleep quality with anxiety disorders, behavioral challenges and psychiatric ailments (37, 38).

Consistent with our first hypothesis, adolescents with moderate and severe food insecurity levels were at higher risks of severe sleep disturbance than those who were food secure. Our findings resonate with prior literature. In a longitudinal study assessing food insecurity among economically challenged households, adolescents in low and moderate-food-insecure households/groups had more sleep disturbances than their counterparts in food-secured households (9). Conterminously, among 223,561 adolescents sampled for a multi-country study on the association between food insecurity and sleep disorders, it was reported that severe food insecurity was significantly associated with a higher risk of sleep disturbance in 48 of the 68 countries studied (11). Transcending the adolescent groups, other studies have reported similar findings among older adults (2) and the general population (10, 13).

Even though the exact mechanisms linking food insecurity (hunger) and sleep disturbances are unclear, some biological and socioeconomic mechanisms may offer plausible explanations. First, considering biological mechanisms, an individual’s metabolic activity may disturb his/her state of vigilance due to lower nutrition, leading to insomnia (39). In addition, chronic stress or psychological distress because of one’s financial ability to access safe and nutritious foods may influence stress related to physiological arousal mechanisms, leading to the instigation of the sympathoadrenal medullary and hypothalamic–pituitary–adrenal-cortical systems that are noted to cause sleep disturbances (10). Besides, adolescents with food insecurity tend to encounter nutritional deficiencies and obesity, which can affect their sleep (40). Similarly, several studies suggest that food insecurity is associated with adverse physical and mental health outcomes that affect sleep (41, 42).

For socioeconomic mechanisms, poverty is a significant risk factor for poor sleep quality (4, 43). While poverty typically does not occur in isolation, with a tight household budget, the family may be unable to pay for food, housing, clothing, health care, and other living expenses simultaneously (43). Consequently, food insecurity is always associated with housing insecurity, and poor housing conditions may link to sleep disturbance due to concerns about personal safety, exposure to more noise from neighbours, and environmental situations within the house (44). This study has a policy implication because addressing food insecurity may be crucial to improve sleep quality and other phycological problems. However, further longitudinal and clinical research is necessary to confirm these hypotheses and explore more complex mechanisms that may underlie adolescent food insecurity and quality sleep.

In line with the differential vulnerability concept, the analysis found significant age-wise differences in the relationship between food insecurity and sleep disturbance. The effect of food insecurity on sleep disturbance was found in adolescents aged 15–17 years and 18 years or older than those between 12 and 14 years. A possible explanation may be due to the socioeconomic conditions of the households, such as poverty and other socioeconomic deprivations, which might cause older adolescents to sacrifice their food intake for younger ones in times of limited household food access. The age differences may be addressed further by decreased brain capacity and functionality as we age, as measured by hippocampal sizes. In the setting of food insecurity, this may raise the likelihood of sleep problems.

Further mixed-method studies would be required to clarify and provide reasons for this result, as this is the first study coming forth with this finding. We found statistically significant sex-wise differences in the relationship between food insecurity and sleep disturbance. This finding agrees with previous propositions that females are less likely than males to achieve their dietary requirements due to general socioeconomic deprivations among females, especially in developing countries (24). Another study on the hidden penalties of gender inequality also found that females likelihood of achieving all the dietary requirements is lower than their male counterparts (45). Our finding, therefore, suggests that boys and girls in the study are disproportionally affected by food insecurity compared to food security. The finding further supports the male–female health survival paradox suggesting that women generally present higher lifetime health problems (46), such as poor sleep and their associated social determinants of health, including food insecurity, than men. Thus, future studies would benefit from further examination of sex differences in the association between food insecurity and sleep disturbances.

### Policy implications

Our study findings suggest that policymakers, guardians, and stakeholders should know that food insecurity (hunger) could be an underlying factor for adolescents’ sleep disturbances. Our results can help facilitate more empirical research to explore sleep disorders within the context of poverty and social inequality, with hunger being a relevant daily stressor that can impact poor sleep outcomes. Evidence exists on interventions addressing the negative impacts of food insecurity in children and adolescents (47). However, such interventions are mostly not multi-faceted to address this area of need (3). For instance, the World Food Programme (WFP) was implemented to support school meal programs in 71 countries in 2017 (48). However, one study reports that the benefits of school-based programs do not address food insecurity holistically as adolescents also require to be fed outside the school environment [for instance, dinner, weekends, holidays, and breaks (3)]. Again, these programs would not benefit those who are not in school. Thus, a long-term solution would be alleviating poverty and acquiring agricultural inputs so that households can potentially meet their dietary needs to address sleep problems. Again, one solution may be free or subsidized nutrition programs that promote access to adequate food for adolescents in food-insecure households. Future interventions and programs to address food insecurity and sleep disturbances should consider demographic factors such as age and sex.

### Strengths and limitations

There are particular strengths and limitations to the present study. Drawing evidence from 35 countries and territories, this study contributes to the limited knowledge base by assessing the association of food insecurity (hunger) with sleep disturbance among school-going adolescents. Our sample used nationally representative datasets with large sample sizes and across several countries and territories, increasing the study findings’ generalizability. The GSHS followed best standard practices regarding technique and employed professional and well-trained interviewers. Notwithstanding these advantages, our findings should be evaluated and viewed in light of the following limitations. Although we controlled for most known potential confounders, residual confounding may exist and affect or explain the results.

Furthermore, reliance on self-reports implies that recollection and social desirability biases cannot be ruled out. In addition, food insecurity is limited to indicating only food insecurity experiences in the last 30 days, while worry-induced sleep disturbance in the previous 12 months indicates a potential timeframe imbalance between the measures. Again, the use of a single-item measure approach is an important limitation in this study because it does not capture all the different dimensions of food insecurity. Future studies are needed to further validate single-item food insecurity measures among adolescents across regions of the world.

Previous studies have established that of those who are currently food insecure, most were food insecure in the past year (4, 11, 32). In addition, notable studies using the same GSHS studies used the past 30 days and 12 months timeframe for the assessment of key variables (4, 49–53). Moreover, given that this was a cross-sectional observational study, causal relationships and implications from the association between food insecurity and sleep disturbance cannot be inferred. Thus, future research should investigate the associations of interest utilizing longitudinal cohort designs that allow for examining reasonable causal inferences.

## Conclusion

This cross-sectional study among adolescent populations from 35 countries and territories showed a significant association between food insecurity and sleep disturbance. Furthermore, age disparities in this association were evident such that females and those aged between 15 and 17 years and 18 years or older experienced higher risks of sleep disturbances than those between 12 and 14 years. Our findings indicate the potential importance of addressing food insecurity (hunger) and social inequality to improve global sleep quality/outcomes among adolescents across countries and world regions. Reverse causation cannot be excluded, and the results must be interpreted cautiously.

## Data availability statement

The original contributions presented in the study are included in the article/supplementary material, further inquiries can be directed to the corresponding author.

## Author contributions

EO, PP, and CA conceived the study. EO, BA, and PP acquired and analyzed the data. EO design the work and the creation of tables. RO, SN, and MA performed the design and drafted the work. All authors contributed to the article and approved the submitted version.
